# Adiponectin Mediated MHC Class II Mismatched Cardiac Graft Rejection in Mice Is IL-4 Dependent

**DOI:** 10.1371/journal.pone.0048893

**Published:** 2012-11-14

**Authors:** Daxu Li, Julia Y. S. Tsang, Jiao Peng, Derek H. H. Ho, Yee Kwan Chan, Jiang Zhu, Vincent C. H. Lui, Aimin Xu, Jonathan R. Lamb, Paul K. H. Tam, Yan Chen

**Affiliations:** 1 Paediatric Surgery Division, Department of Surgery, The University of Hong Kong, Hong Kong SAR, China; 2 Department of Chemistry, The University of Hong Kong, Hong Kong SAR, China; 3 School of Biological Science, The University of Hong Kong, Hong Kong SAR, China; 4 Department of Medicine, The University of Hong Kong, Hong Kong SAR, China; 5 Division of Cell and Molecular Biology, Faculty of Natural Sciences, Imperial College London, London, United Kingdom; University of Florence, Italy

## Abstract

**Background:**

Adiponectin regulates glucose and fatty-acid metabolism but its role in chronic graft rejection mediated by Th2 cytokines remains ill-defined.

**Methodology/Principal Findings:**

Wild type and adiponectin-null mice were used as graft recipients in mouse MHC class II disparate cardiac transplantation (bm12 toB6) and the graft rejection was monitored. In adiponectin-null mice we observed that the cellular infiltrate of eosinophils, CD4^+^ and CD8^+^ T cells was reduced in grafts compared to the controls as was collagen deposition and vessel occlusion. A similar outcome was observed for skin transplants except that neutrophil infiltration was increased. Low levels of IL-4 were detected in the grafts and serum. The effect of adiponectin signaling on IL-4 expression was further investigated. Treatment with AMPK and p38 MAPK inhibitors blocked adiponectin enhanced T cell proliferation in mixed lymphocyte reactions. Inhibition of AMPK reduced eosinophil infiltration in skin grafts in wild type recipients and in contrast AMPK activation increased eosinophils in adiponectin-null recipients. The addition of adiponectin increased IL-4 production by the T cell line EL4 with augmented nuclear GATA-3 and phospho-STAT6 expression which were suppressed by knockdown of adiponectin receptor 1 and 2.

**Conclusions:**

Our results demonstrate a direct effect of adiponectin on IL-4 expression which contributes to Th2 cytokine mediated rejection in mouse MHC class II histoincompatible transplants. These results add to our understanding of the interrelationship of metabolism and immune regulation and raise the possibility that AMPK inhibitors may be beneficial in selected types of rejection.

## Introduction

In mammals body metabolism and immune function are tightly linked. Adiponectin (APN) is an adipokine mainly produced by adipocytes and functions in glucose and fatty-acid metabolism but it has also been shown to have anti-inflammatory activities [1]. The mechanism was initially attributed to up-regulated IL-10 [2]. Later, work on human macrophages suggested many other factors are involved including A20 and SOCS-3 but not IL-10 [3]. This difference may be due to the various isoforms of APN in that globular adiponectin functions via IL-10 and HO-1 pathways while full length APN uses the IL-4/STAT6 pathway to polarize macrophages to an M2 phenotype [4]. APN can also modulate adaptive immunity. Two adiponectin receptors AdipoR1 and AdipoR2 are present in both human and mouse derived T cells and ligation of these receptors has inhibitory effects on activated T cells [5], [6]. Bone marrow derived dendritic cells (DC) treated with APN showed only moderate differences in differentiation but their function was altered such that they reduced the proliferative capacity of allogeneic T cells and enhanced regulatory T cell production [7]. In animal models of fully mismatched MHC class II heart transplantation in which the Th1 cytokines play dominant role increasing APN expression with a PPAR-γ agonist reduced graft rejection and prolonged survival [8], [9]. However, the role of APN in regulating Th2 dominant rejection remains to be clarified.

**Figure 1:**
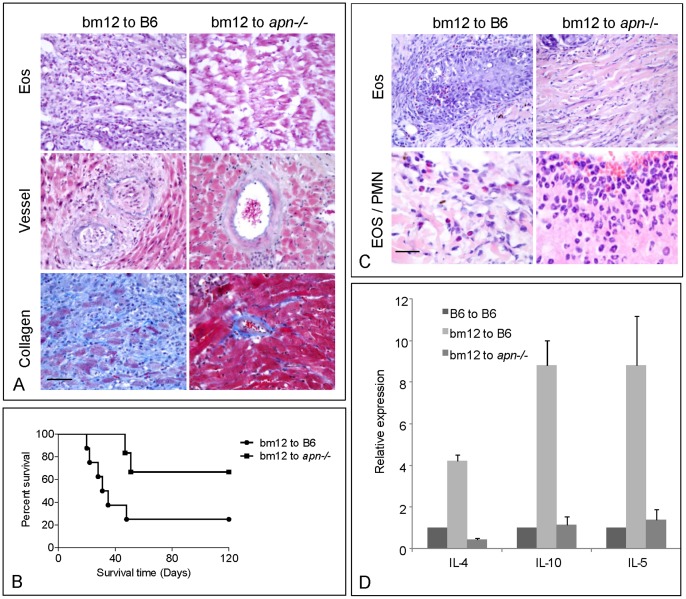
Effect of adiponectin in mouse MHC class II mismatched cardiac and skin transplantation. A. Heterotopic cardiac transplantation with bm12 mice as donors, B6 (left panel, n = 6) and Apn–/– (right panel, n = 5) as the recipients. The grafts were examined after the heart beat had ceased. The tissue sections were stained for eosinophils (Eos), vessel occlusion and tissue fibrosis. B. Cardiac graft survival curves in bm12 mice to B6 or Apn–/– mice. C. Skin transplantation was performed and the tissues were collected at 7 days after operation. The sections were stained and analyzed. For observation of neutrophil infiltration, the high magnification (1,000 x) was used in HE stained section (Eos/PMN left) in compared with eosinophils (Eos/PMN right). D. Cytokines mRNA expression in heart grafts. The expression of cytokines was measured with quantitative-PCR. One comparable sample each was used for the syngeneic group (B6 to B6), wild type group (bm12 to B6) and Apn–/– group (bm12 to Apn–/–) and the data were present as triplicates for each measurement. Scale Bar = 50 µm.

Mouse MHC class II mismatched cardiac transplantation with bm12 to B6 as donor and recipient is a model often used to study chronic rejection. The absence of alloreactive CD8^+^ T cells biases immune rejection to a Th2 phenotype [10]. IL-4 is central in initiation of the response since IL-4 deficient mice or the administration of an IL-4 neutralizing antibody reduces graft rejection and promotes long-term survival [11], [12]. Using this model we have reported that the PPAR-γ agonist rosiglitazone increased eosinophil infiltration in heart transplants [5] suggesting that in this type of graft rejection APN plays a different role.

In this study, with the *apn*−/− mice (B6 background) as recipients the immunomodulatory effects of APN have been investigated in detail both *in vitro* and *in vivo*. There was no chronic rejection, cellular infiltration was minimal and IL-4 expression was reduced in *apn*−/− compared to wild type mice. In mixed lymphocyte reactions (MLRs) the presence of APN enhanced T cell proliferation which was reduced by anti-IL-4 antibody treatment. GATA-3 and p-STAT6 were induced by APN but the induction was blocked by siRNAs specific for APN receptor 1 and 2. Our data here show a direct relationship between APN and IL-4 expression and illustrate cross talk between metabolic pathways and immune regulation.

**Table 1:** Cellular infiltration, vasculopathy and tissue fibrosis in heart and skin grafts.

Donor: bm12	Heart transplant		Skin transplant	
Recipient:	B6	*Apn–/–*	B6	*Apn–/–*
CD4	36±2	6±1***	20±2	0***
CD8	11±1	1±0***	30±2	0***
Eos	79±4	0***	26±2	0***
Vessel occlusion (%)	99.5±0.5	7.95±5.8***	ND	ND
Collagen deposit (%)	28.5±2.9	3.5±0.4***	ND	ND

ND: No determined. ***p<0.001 compared to B6.

## Materials and Methods

### Animals

Inbred male C57BL/6 (B6, H-2^b^), B6.C-H-2^bm12^KhEg (bm12, H-2^bm12^), and BALB/c (H2d) mice (6–8 weeks old, weight of 20–25 g) were maintained in the Laboratory Animal Unit, University of Hong Kong. Adiponectin knockout (*apn*–/–) mice were generated in C57BL/6J background as described previously [13]. The serum APN level was examined by ELISA, in *apn*–/– mice it was undetectable compared to 16.5±3.10 µg/ml in B6 mice (*apn+/+*) and in 4.1±0.03 µg/ml heterozygous (*apn+/–*). All experimental protocols were approved by the Committee on the Use of Live Animals in Teaching and Research, University of Hong Kong (Ref: 1591-07).

**Table 2:** Serum levels of IL-4 at 7 days post-operatively in mice following skin grafting.

	UT	CompC	MET
B6 to B6	8.36±2.83	ND	14.0±0.52*
*Apn*–/– to *Apn*–/–	0.70±0.27†	ND	4.57±1.25**
bm12 to B6	36.5±7.23†	14.3±0.68‡	ND
bm12 to *Apn*–/–	1.54±0.37	ND	10.6±3.61**

UT: untreated; ND: not determined. n = 6 in each group (†*p*<0.01 compared to B6 to B6; ‡*p*<0.001 compared to UT; *p<0.05 and **p<0.01 cpmpared to UT).

### Reagents and Chemicals

Reagents and chemicals used are described in the Materials S1.

### Heterotopic Cardiac Transplantation and Skin Transplantation

Mouse cardiac transplants were performed as previously described[5] using heterotopic cardiac allografts from bm12 mice as donors and C57BL/6 (B6) mice or *Apn*–/– mice as recipients. Standard microsurgical techniques were used for vascularized grafts transplantation. In brief, the donor heart was carefully detached from the thoracic cavity and placed in cold Lactated Ringers solution containing 100 U/ml heparin. The time for the donor operation was less than 20 min. The donor ascending aorta and the pulmonary trunk from the heart graft was anastomosed end-to-side to the recipient abdominal aorta and inferior vena cava, respectively. The time required for revascularization of aortotomy and venotomy was < 50 min. The animals were observed daily and the loss of viability of the grafts was determined by cessation of palpation and confirmed by histological analysis. Skin transplantation was performed as described previously [14]. Briefly, full-thickness donor skin was grafted to the recipients and the graft sites were covered by plaster removed on day 7. Grafts were observed daily and the rejection was determined when no viable skin remained.

**Figure 2:**
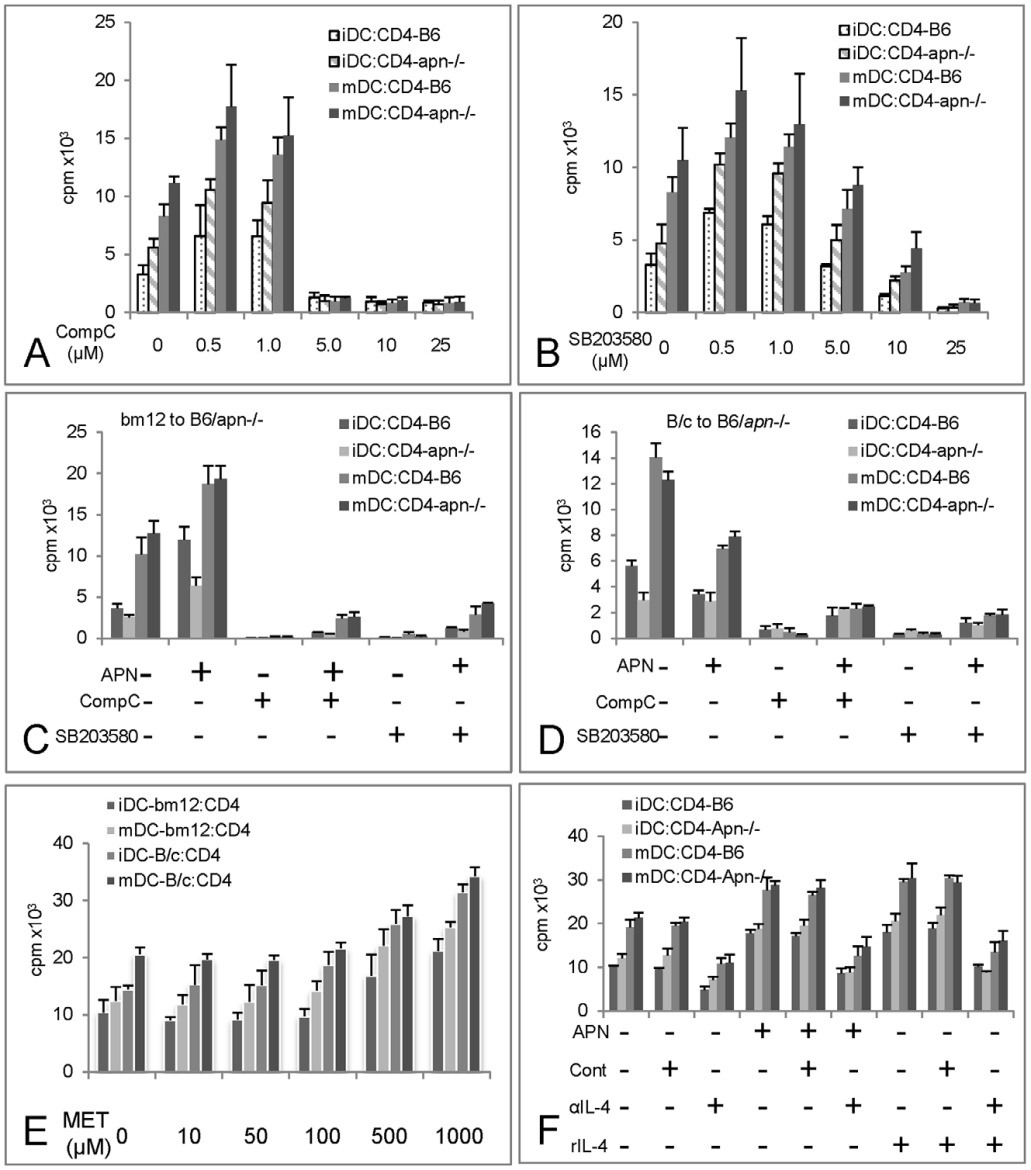
Effects of adiponectin and its signaling pathways on dendritic cell (DC) induced T cell function. Bone marrow derived dendritic cells from bm12 mice were used as stimulator cells and CD4+ T cells from B6 or Apn–/– mice as responder cells. Cell proliferation was measured by ^3^H-thymidine incorporation. Immature DCs were treated with LPS for an additional 24 h to induce maturation. n = 4 and one representative graph is presented. The APN signaling inhibitors, Compound C (CompC) for AMPK (A) and SB203580 for p38 MAPK (B) were added at different concentrations. The effect of APN and signaling inhibitors on T cell proliferation in MLRs of DCs from bm12 and T cells from apn–/– was measured. APN (5 µg/ml) was added to MLRs for 24 h in the presence or absence of CompC or SB203580 (C). Similarly the compounds were tested in MLRs with DCs from Balb/c (B/c) mice (D). Effect of Metformin (MET) on T cell proliferation was assessed. DCs were obtained from either bm12 or B/c and the T cells were isolated from B6. MET was added to MLRs and the T cell proliferation was measured as above (E). The effect of IL-4 on APN induced proliferation was evaluated. APN (5 µg/ml), anti-IL-4 antibody (αIL-4, 50 µg/ml) and mouse recombinant IL-4 (rIL-4, 50 ng/ml) were added to the MLRs and T cells proliferation were measure as described as above (F).

### Histological and Immunohistochemical Analysis of Grafts Rejection

Both paraffin and frozen sections (4 µm) were analyzed. The morphology was observed with haematoxylin and eosin (HE) staining. CD4^+^ and CD8^+^ cells were detected by immunohistochemical staining. Carbol-chromotrope, Victoria blue and Masson’s trichrome staining were used to visualize of eosinophil infiltration, blood vessel elastin and tissue collagen deposits respectively and the counting methods used were as published [5].

**Figure 3:**
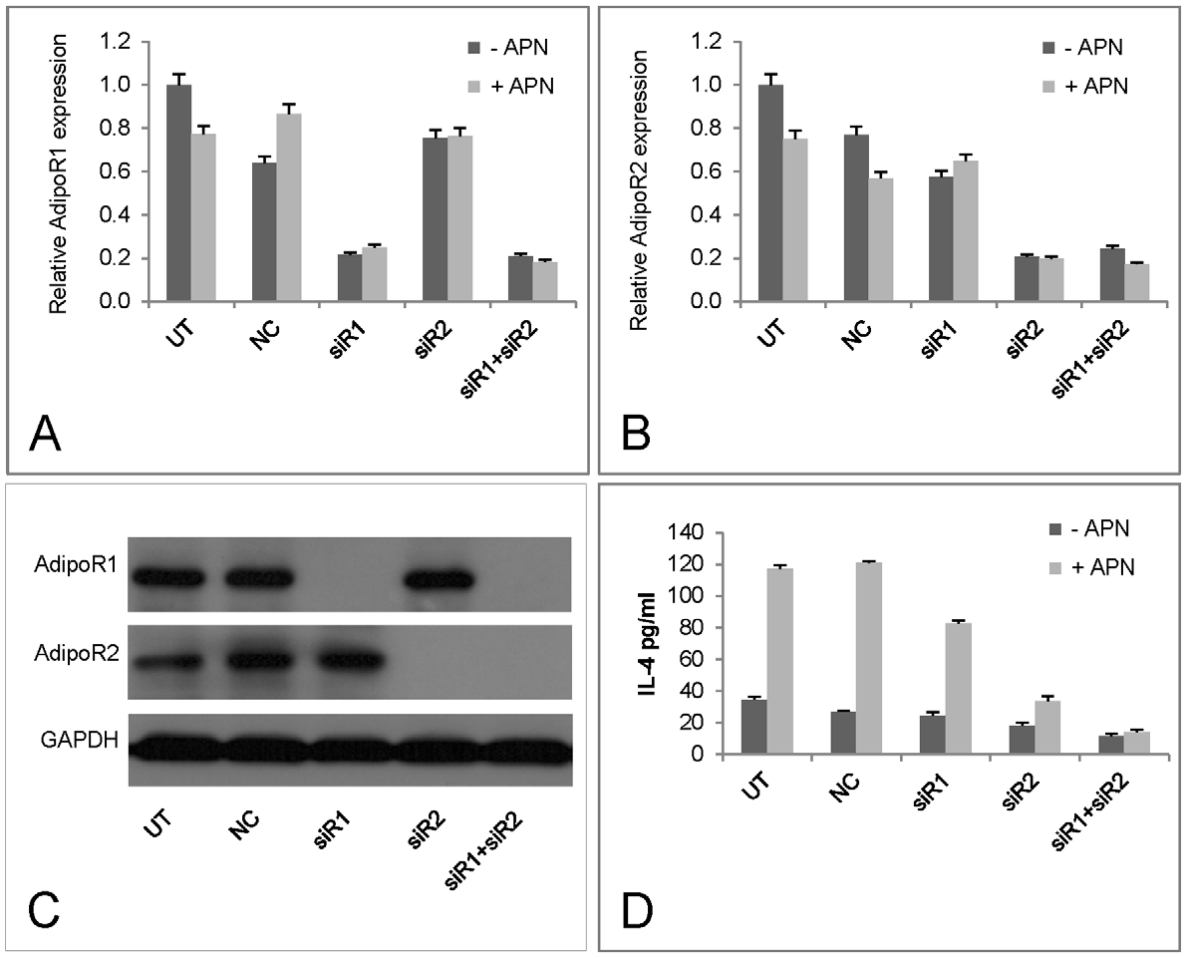
IL-4 production in response to adiponectin receptor AdipoR1 and AdipoR2 knockdown using siRNA. EL-4 cells were transfected with siRNA for AdipoR1 and AdipoR2 for 24 or 48 hours together with control siRNA (n = 2). The effects of siRNA were detected both with Q-PCR and Western blot with specific antibodies. Supernatants were collected for the measurement of IL-4 production.

### Serum Level of Cytokines Expression

Serum and supernatants were collected and stored at −80°C. Cytokine levels were measured by standard ELISA methods following the manufacturer’s instructions.

### Mixed Lymphocyte Reactions

Splenocytes from bm12 and BALB/c mice were used as stimulators and B6 or *Apn*–/– mice as responders. To reduce the effect of adiponectin present in serum the volume of fetal bovine serum added to cell proliferation assays was sequentially diluted and we observed that the addition of 0.5% FBS had minimal effects and therefore was used at that volume in the subsequent experiments (Fig S4 A) Responder splenocytes (1×10^6^) were co-cultured with irradiated stimulating cells at a 1∶1 ratio in triplicate for 2 days before the ^3^H-thymidine was added. The cells were cultured for 1 more day and the cell proliferation was measured using a beta counter (PerkinElmer. Inc, MA USA).

**Figure 4:**
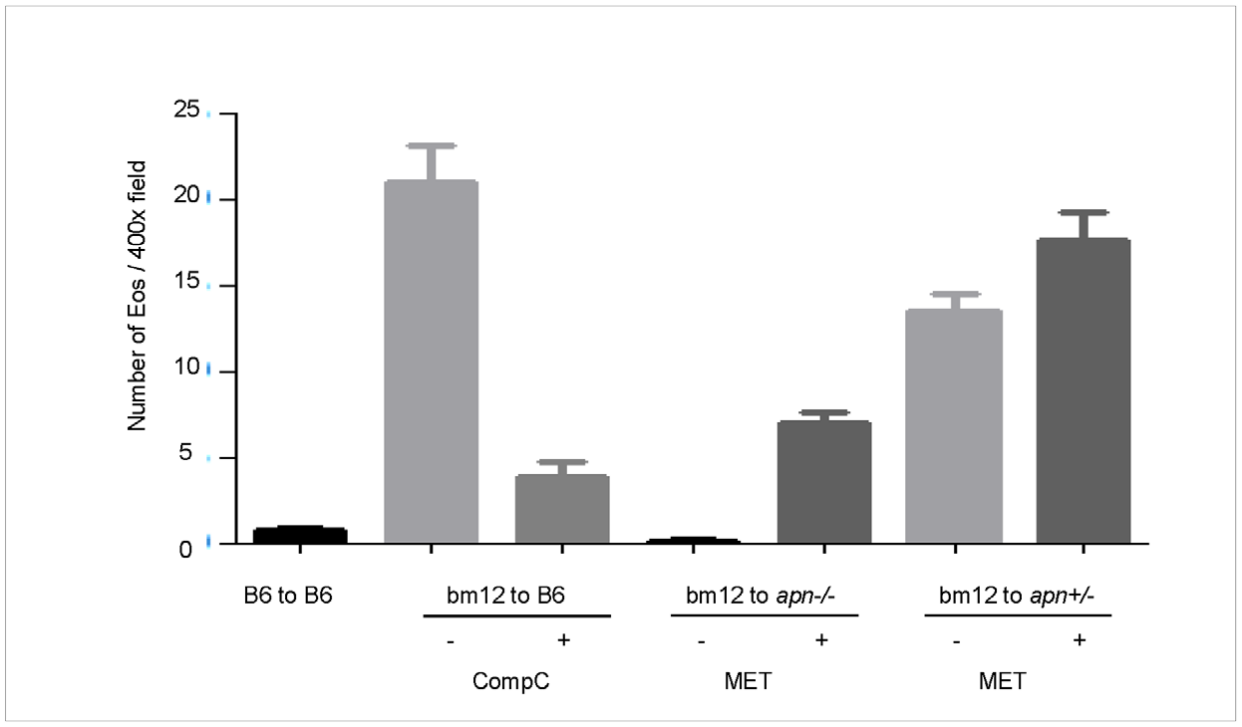
Effect of adiponectin signaling pathway inhibitors and agonist on MHC class II mismatched skin transplantation. For skin transplantation bm12 mice were used as donors and B6, Apn–/– or APN+/- mice as recipients (n = 6 each). The animals were treated with Compound C (CompC, 5 mg/kg/day), Metformin (MET, 250 mg/kg/day) and the grafts were obtained at day 7 after transplantation. The number of eosinophil was counted the average is shown.

### *In vitro* Generation of Dendritic Cells and T Cells Proliferation Assay

DCs were generated from bone marrow using the protocol of Steinman *et al*. with minor modifications.[14] In brief, bone marrow cells were negatively isolated and cultured with granulocyte-macrophage colony-stimulating factor (GM-CSF) for 7 days. Maturation of DCs was induced by adding 100 ng/ml of lipopolysaccharide (LPS) on day 6 to cultures of immature DCs which were then incubated for an additional day. Dynal® CD4 Negative Isolation Kit was used to isolate CD4^+^T cells from B6 or *Apn*–/– spleens. CD4^+^T cells (1×10^5^cells/well) were stimulated with γ-irradiated (30 Gray) DCs from bm12 mice in 96 well plates. Proliferation was assessed by ^3^H-thymidine incorporation during the last 24 hrs of 3 day cultures. To determine the amount of IL-4 produced by T cells supernatants were harvested on day 1 of the DC-T MLRs. The AMPK and P38 inhibitors (compound C and SB203580) were dissolved in DMSO and the final volume of DMSO was <0.1%. Their ability to induce apoptosis was tested for by Annexin V staining and sub-apoptotic concentrations were then used the subsequent experiments.

**Figure 5:**
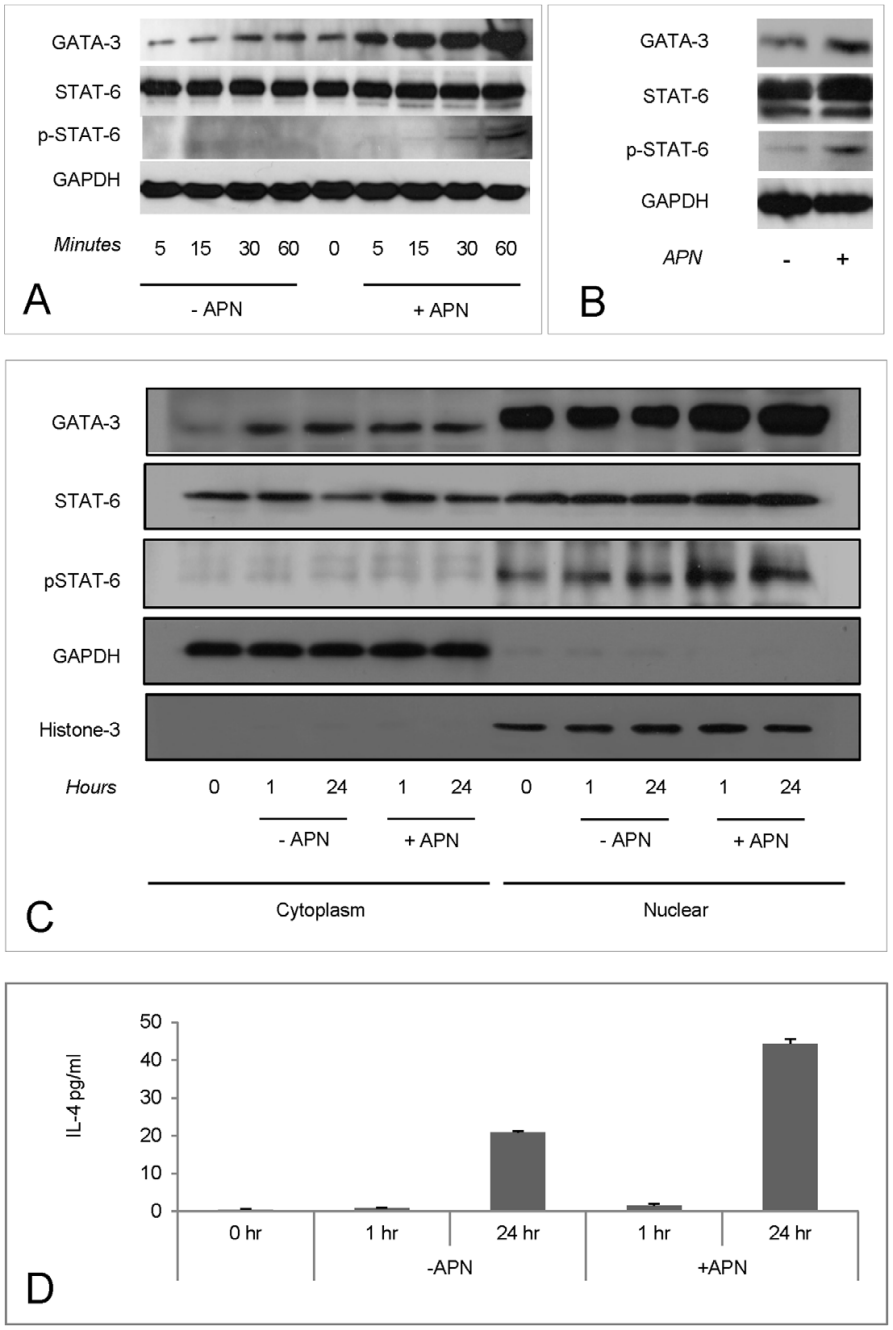
GATA-3 and STAT6 expression in response to Adiponectin (APN). Intracellular signaling pathways for IL-4 production were detected using the IL-4 producing T cell line EL-4. Cells were cultured in RBMI medium with 2.5% fetal bovine serum. The APN (5 µg/ml) was added and the samples were collected with the indicated time points (n = 3). The expression of GATA-3 and STAT6 was detected in whole lysates (A and B) or separated cytoplasmic and nuclear parts (C and D) using Western blot. The expression of IL-4 was detected in supernatants with ELISA.

### Quantitative-PCR for Analysis Genes Expression Level

The mRNA expression levels were examined using the GoScript™ Reverse Transcription System and quantitative real-time PCR (Q-PCR) with SYBR® Green-Based Detection system. The primers sequences were showed in Table S1. Relative quantification of mRNA expression was normalized with control GAPDH and analyzed by the Delta Delta Ct (2^−ΔΔCT^) method.

### Protein Expression Examination with Western Blot

The protein expression levels were determined by Western blotting. 30 µg of protein was denatured and separated by electrophoresis. After electro-transferred onto PVDF-membranes and blocking with 5% low-fat milk TBST, the primary antibodies were added and incubated overnight at 4°C and then incubated with horseradish peroxidase (HRP)-conjugated secondary antibody. The signals were developed using ECL chemi-luminescence (Amersham). The membranes were stripped and reprobed with an anti-actin antibody. Rat anti-mouse GATA-3 (1∶1000), rabbit anti-mouse p38 (1∶1000) and p-p38 (1∶1000), rabbit anti-mouse STAT6 (1∶1000) and p-STAT6 (1∶500), goat anti-mouse GAPDH (1∶5000), rabbit anti-mouse Histone3 (1∶5000), rabbit anti-mouse AdipoR1 and 2 (1∶500) were used in experiments.

**Figure 6:**
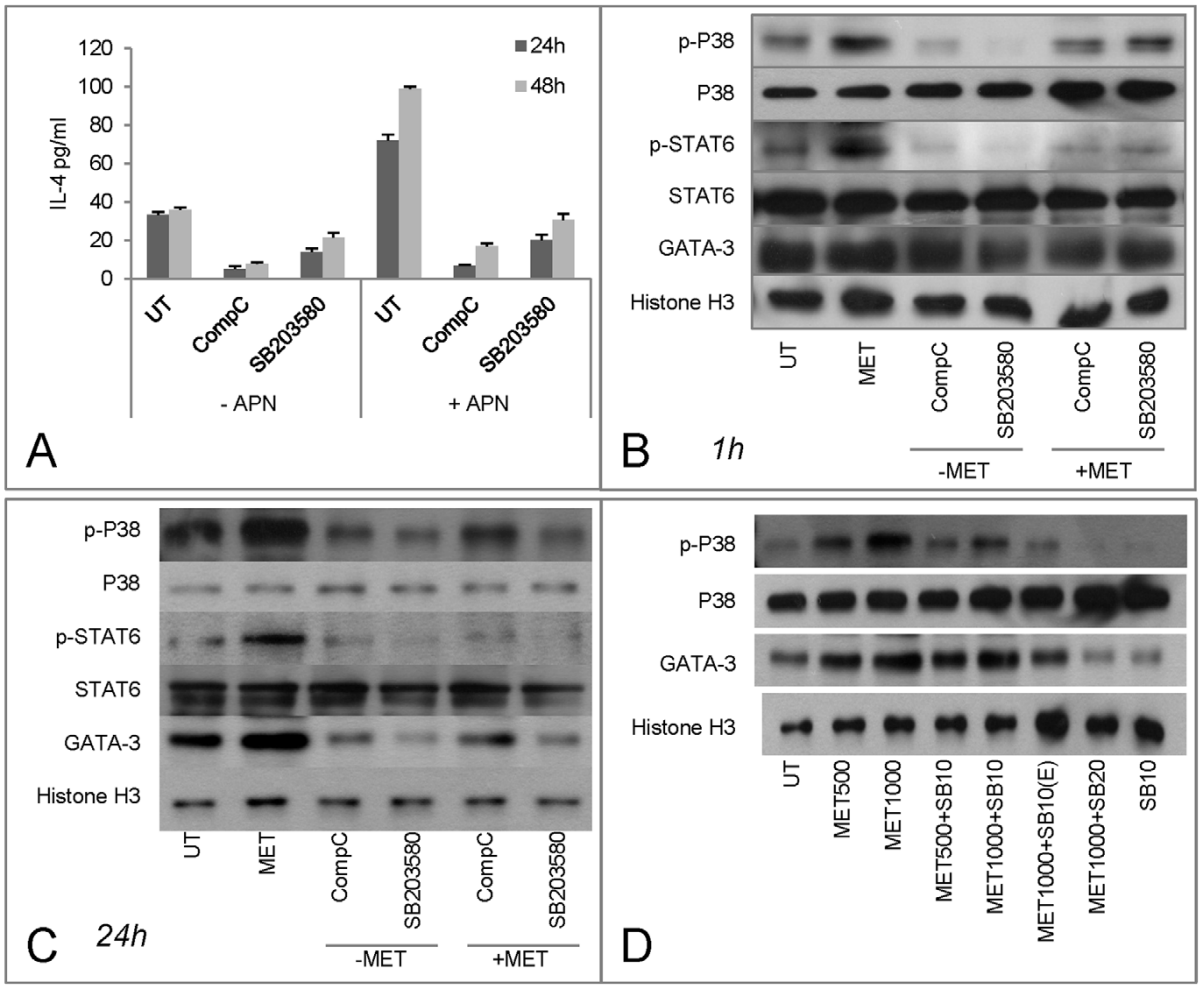
Functions of AMPK and p38 in IL-4 expression in response to Metformin (MET) treatment. A. EL-4 cells were cultured with APN (5 µM), Compound C (CompC, 10 µM) and SB203580 (10, 20 µM) for 1 h and 24/48 h and supernatants were collected for IL-4 detection (n = 3). B and C. Nuclear proteins were obtained for intracellular signaling pathway molecules detection using Western blots. D. MET induced up-regulation of p-p38 was inhibited by increasing the dose of SB203580 or reducing that of MET or by pre-treating with SB203580 for 1 h (E) by Western blots.

### Inhibition of Gene Expression with siRNAs

Specific synthetic siRNAs were obtained from Invitrogen Hong Kong Ltd. Lipofectamin was used to transfect cells following the manufacturer’s instructions. In brief, 2×10^5^ EL4 cells were cultured to 50% confluence before the RNAi duplex-Lipofectamine™ RNAiMAX complexes were added. The transfection was repeated after 24 hours. The success of inhibition effect was measured by quantitative (Q)-PCR analysis with AdipoR1 and AdipoR2 primers (Table S1) and further confirmed by Western blotting.

### Statistical Analysis

Kaplan-Meier method and the log-rank test were used to analyze graft survival. Mann Whitney test or Dunn’s Multiple Comparison Test were used to compare two or three sets data respectively. *p*<0.05 was considered significant. All cell cultures were performed in triplicate and error bars represent the standard error of mean (SEM). Statistical analysis was performed with Prism software (GraphPad Software, La Jolla, CA).

## Results

### Absence of Chronic Rejection in bm12 to apn–/– Heterotopic Transplants

In bm12 to B6 mice heterotopic cardiac transplants extensive eosinophil infiltration is a hallmark of chronic rejection. Compared to wild type controls (B6) when *apn*–/– mice were the recipient only minimal cellular infiltration was observed (Fig. 1A). CD4^+^ and CD8^+^ T cell infiltration was reduced by 90% compared to bm12 to B6 grafts and no eosinophils were observed in graft tissue sections (Fig. 1A, Table1 and Fig. S1). Blood vessel occlusion was also significantly reduced. Similarly, collagen deposition was 8 fold reduced (Fig. 1A and Table1). Concomitantly, graft survival was prolonged with the median survival time of 33 days increasing to >120 days. The mean rejection time was extended from 31 days to 49 days and only 2/8 WT mice survived >120 days compared to 4 of 6 *apn*–/– mice (Fig. 1B). In skin transplants at 7 days, minimal numbers of eosinophils, CD4^+^, and CD8^+^ T cells were detected in the grafts in *apn*–/– compared to B6 mice (Fig. 1C, Table1 and Fig. S2), whereas in the *apn*–/– mice a larger number of neutrophils were present in the infiltrate (Fig. 1C).

### Expression of Cytokines in Allografts and Serum

Chronic rejection in MHC class II histoincompatible transplantation is driven by Th2 cytokines with IL-4 expression key to the initiation of the immune response. IL-4 expression in both grafts and serum were examined by Q-PCR and ELISA. Compared to B6 transcripts for the Th2 cytokines IL-10, IL-4 and IL-5 were reduced in the *apn*–/– mice (Fig. 1D). In serum of skin transplant recipients at days 7 post-operatively low levels of IL-4 were found in *apn*–/– to *apn*–/– grafted mice. IL-4 production was increased 4 fold in B6 recipients compared to syngeneic group, but only 2 fold in *apn*–/– recipients (Table 2), suggesting that IL-4 up-regulation in *apn*–/– mice in response to skin grafts might be dysregulated. We also observed that mRNA expression of the Th1 cytokines, IL-2 and IFN-γ in grafts was reduced in the *apn*–/– compared to B6 recipients. Furthermore, IL-17 and PD-L1 were reduced in the cardiac transplants whereas expression of the APN receptors AdipoR1 and AdipoR2 was increased in *apn*–/– (Fig S3). Serum levels of Th1 cytokines showed no significant difference although slight increase of IFN-γ was observed (Table S2).

### Effect of Inhibition of APN Signaling on Mixed Lymphocytes Reactions (MLRs)

To test the effect of inhibiting APN signaling on T cell proliferation in B6 or *apn*–/– mice, bone marrow derived DCs from bm12 mice were cultured for 7 days and then treated with LPS to induce DC maturation (Fig. S5B and 5C). For comparison, DCs from BALB/c (B/c) mice were used in some experiments. T cells isolated from B6 or *apn*–/– spleens were used as responder cells. Both immature and mature DCs were analyzed in MLRs at DC to T cell ratios of 1∶10 (Fig. S5A). Dose dependent inhibition with both CompC and SB203580 was observed with CompC being the more potent inhibitor which at 5 µM completely inhibited T cells proliferation but 25 µM in SB203580. At these concentrations SB203580 induced apoptosis but CompC did not (Fig S4 B & C). Mature DCs significantly increased the proliferation of both B6 and *apn*–/– derived T cells. Although a higher response to bm12 DCs stimulation was induced in *apn*–/– compared to B6 T cells there was no statistical significant between the two (*p*>0.05; Fig. 2A.and 2B).

APN alone amplified the proliferative response of both B6 and *apn*–/– T cells to bm12 DCs which was inhibited by the treatment with CompC or SB203580 (Fig. 2C). The addition of APN to MLRs with BALB/c derived DCs as stimulator cells inhibited T cell proliferation (Fig. 2D). CompC and SB203580 could not fully inhibit proliferation in the present of exogenous APN and their inhibitory were only partially reversed by APN.

As APN signals mainly through AMPK we further investigated if enhancement of AMPK activity modulated T cells proliferation. Metformin (MET) is an AMPK agonist and addition of this compound induced a dose dependent increase in T cell proliferation in B6 and *apn*–/– T cells but with no obvious difference between the two. At doses of 500 µM (*p*<0.05) and 1000 µM (*p*<0.001) the increase in T cell proliferation was significant but was similar both in B6 and in *apn*–/– groups (Fig. 2E).

As the effect on T cell proliferation might relate to IL-4 production an anti-IL-4 neutralizing antibody was added. The anti-IL-4 antibody alone effectively inhibited bm12 induced T cell proliferation when either immature DC or mature DCs were present in the cultures (*p*<0.01). APN enhanced T cell proliferation was also suppressed by the anti-IL-4 antibody when either immature DC or mature DCs were used as stimulator cells (*p*<0.01 and 0.001 respectively), suggesting that proliferation was partially mediated by IL-4 (Fig. 2F).

### The Effect of Target Specific Inhibition of APN Signaling on IL-4 Production

To investigate the contribution of APN signaling on IL-4 production specific siRNAs for APN receptors AdipoR1 and AdipoR2 were used and the outcomes measured by Q-PCR and protein expression. Compared to control or irrelevant siRNAs, which induced a maximum of 24% reduction in receptor expression in the presence of APN, specific siRNAs for AdipoR1 and AdipoR2 resulted in 65 to 73% inhibition. When the two siRNAs were added together expression of AdipoR1 and AdipoR2 mRNA was inhibited >80% (Fig. 3A and 3B). Modified AdipoR1 and AdipoR2 protein expression was confirmed by Western blotting (Fig. 3C). Specific inhibition of AdipoR1 and AdipoR2 reduced APN induced IL-4 production by 31.7 and 72.2% respectively, in case of double inhibition, 88.5% of APN induced IL-4 production was suppressed (Fig. 3D). Thus APN plays an important role in IL-4 production directly through its action on AdipoR1 and R2 with activation of the latter being more efficient.

### Effect of Modulating APN Signaling on bm12 to B6 or apn–/– Skin Transplantation

Since targeting APN could inhibit or enhance T cell proliferation and IL-4 production *in vitro* we extended these studies to determine possible *in vivo* effects on the survival of skin grafts. Eosinophil infiltration is characteristic of the immune response in bm12 to B6 skin grafts therefore we evaluated the number of eosinophils in bm12 grafts in B6 or *apn*−/− mice treated *in vivo* with CompC or MET (Fig 4). On day 7 the number of eosinophils was greatly reduced following treatment with CompC compared to untreated control group with B6 mice as the recipients (4±3 vs. 21±9, *p*<0.001), whereas eosinophil infiltration was reversed in bm12 grafts in *apn*–/– recipients receiving MET (7±3 vs. 0, *p*<0.01). These results were confirmed using *apn*+/– heterozygote mice in which the number of eosinophils was lower than in B6 (*p*>0.05) but higher than in *apn*–/– (*p*<0.001) recipients and eosinophil infiltration was further increased following treatment with MET (Fig. 4). IL-4 production was also measured in B6 recipients treated with CompC and a 61% reduction in serum IL-4 was observed. When compared to *apn*–/– as recipients treatment with MET increased IL-4 in serum by 5.8 fold these effects of MET on IL-4 production were also observed in syngeneic group (Table 2).

### APN Treatment Increased Nuclear GATA-3 and p-STAT6 Expression Levels

To explore further APN effects on IL-4 production expression levels of GATA-3 and STAT6 were measured in the IL-4 producing T cell line, EL4. In the presence of APN the expression of GATA-3 in whole cell lysates was increased within 60 min and maintained at 24 h. No induction of STAT6, which is constitutively expressed, was observed (Fig. 5A and B) however there was a marginal increase in phosphorylated-STAT6 (p-STAT6) at 1 h which was more obvious at 24 h (Fig. 5B). GATA-3 was mainly located in nucleus and its expression was increased by APN treatment. STAT-6 could be found both in cytoplasm and nucleus and nuclear expression of p-STAT6 was enhanced by APN (Fig. 5C). APN treatment increased the level of IL-4 expression in EL4 cell supernatants (Fig. 5D).

### Relationship of AMPK and p38 MAPK in IL-4 Production

APN induced IL-4 expression in EL4 cells was inhibited by CompC and SB203580 suggesting both APN activated AMPK and p38 influence IL-4 production (Fig. 6A). Based on studies in skeletal muscle cells it seems that p38 is a downstream target of AMPK [15], whether or not this is the case for T cells is not clear. Treatment with MET increased phosphorylated p38 (p-p38) levels in the nuclear protein extracts both at 1 h and 24 h time points. Enhanced expression of p-p38 was inhibited by CompC treatment implying that the effect was mediated by AMPK (Fig. 6B and 6C). However, at 1 h SB203580 had no inhibitory effect on p-P38 but at 24 h the effect had a similar magnitude to that of MET. This was confirmed by the increasing the dose of SB203580 or lowering the dose of MET with same dose of SB203580 (Fig. 6D). These results suggest that the effect of APN mediated AMPK induction of IL-4 might be through p38 activation.

## Discussion

In this study we have investigated APN signaling in the regulation of IL-4 using mouse models of MHC class II mismatched transplantation in which the alloreactive immune response is initiated by IL-4 and dominated by Th2 cytokines. When *apn*−/− (B6) mice were used as recipients for bm12 cardiac grafts the survival was increased and accompanied by reduced IL-4 in the grafts and serum. The findings reported here advance our understanding of adiponectin in DC and T cell interactions in Th2 cytokine dominant environments. Low APN levels reduced IL-4 expression which is consistent with previous observations that *apn*−/− mice M1 macrophages are expanded at the expense of M2 cells, which are dependent on IL-4 for their differentiation [16]. In DSS induced intestinal inflammation, the absence of APN reduced tissue damage also similar to that in IL-4 deficiency mice [17] again implying a role for APN in IL-4 production. Furthermore, the upregulation of APN signaling by MET increased eosinophils in skin transplants which supports our previous observation that the PPAR-γ agonist rosiglitazone promotes eosinophil recruitment in heart grafts and suggests that reducing APN could be beneficial in graft survival in MHC class II mismatched grafts rejection. We also noted that chronic rejection in our model (bm12 to C57BL/6) was more pronounced than some previous reports in the literature [18] which might in part relate to the genetic background of the mice used in our study. The C57BL/6 both wild type and *apn*−/− are derived from C57BL/6N and the bm12 obtained from Jackson Laboratory was on a C65BL/6J background. In 6J to 6N strain combination of skin transplantation, a 2 fold increase in IFN-γ and IL-2 in the serum was observed in wild type as well as *apn*–/– compared to the syngeneic controls (Table S2) but there was no significant difference between chronic rejection groups. However, in comparison levels of IL-4 were altered (>4 fold increase in the wild type group, Table1). These observations demonstrate that genetic background can modify qualitative aspects of responses in the same disease model [19]. Our results on graft rejection are contrary to those in which *apn*–/– mice were also used as transplant recipients [20] and this may be due to variations between different strains of *apn*–/– mice as suggested, for example, by the differential response of *apn*–/– mice to DSS induced intestinal inflammation [21], [22]. The underlying differences between of these *apn*–/– mice remain unclear but may relate to the knockout procedure in gene manipulation and/or background as illustrated by the variations in their insulin resistance and glucose intolerance [23].

T cells from *apn*–/– and wild type B6 mice exhibited similar *in vitro* proliferative responses to bm12 splenocytes or bone marrow derived DCs indicated no defect in T cell development or activation. APN enhanced T cell proliferation in bm12 to B6 or *apn*–/– combinations but reduced proliferation when BALB/c derived cells were used as the donor demonstrating the presence or absence of APN is a key factor in T cell proliferation in the MHC class II disparate or fully mismatched combination. The marginally higher T cell proliferation and IL-4 production in *apn*–/– cells to bm12 stimulation is due to elevated expression of the APN receptors especially AdipoR2 (Fig S6). The loss of grafts in *apn*–/– in the absence of alloreactivity suggests that tissue repair is deficient due to ischemia reperfusion, which causes myocardium damage in *apn*–/– mice [24].

One of APN intracellular signaling pathways is p38 [15]. Here we observed a dose dependent of inhibition of T cell proliferation in B6 and *apn*–/– mice with SB203580. CD28 mediated T cell proliferation was through p38 [25] and B7-CD28 co-stimulation is essential in immune rejection of bm12 and B6 combinations [26]. Our data confirmed previous observations that inhibition of p38 reduces T cell proliferation in MLRs of bm12 to B6 [25]. AMPK is another downstream target of APN. Inhibition of T cell proliferation by an AMPK inhibitor was observed in MLRs of bm12 or BALB/c to B6 or *apn*–/– as donors and recipients respectively. In the presence of APN the inhibitory effect of the AMPK inhibitor was incomplete indicating that APN may have an effect in restricting the inhibition effect of CompC. Unlike the direct effects of p38 on antigen presentation it appears that AMPK activation is not essential for activation and expression of effecter molecules following immune stimulation but for their response to metabolic stress [27]. However, others report that AMPK knockdown modulates DC maturation through TLR related metabolic changes [28] which highlights the complexity of AMPK signaling in immune responses need to further investigate.

Regarding IL-4 expression inhibition of AMPK reduced basal levels of IL-4 whereas treatment with the AMPK agonist Metformin enhanced IL-4 production. Metformin also increased phospho-p38 and the effect was further rescued by AMPK inhibition implying that MET induced p38 activation was AMPK dependent and the effect of MET on IL-4 production was through p38 pathway. At higher doses or by pre-treating cells SB203580 had an inhibitory effect of GATA-3 and p-STAT6 confirmed that p38 was involved in MET induced IL-4 expression through AMPK.

In conclusion, our data provide direct evidence that APN aids in promoting IL-4 production that can enhance eosinophil infiltration and contribute to graft rejection and therefore treatment with an APN inhibitor promotes graft survival. The application of APN inhibitors in other Th2 cytokine dominant immune reactions remains to be investigated.

## Supporting Information

Figure S1 **Immunohistochemical analysis of cardiac transplantation grafts.** Sections were stained with Hematoxylin and Eosin (HE), rat anti-mouse CD4 and CD8 antibodies and bm12 to B6 grafts are compared to bm12 to APM−/− grafts. The samples were obtained from the grafts which the heart beating was stopped.
(TIF)

Figure S2 **Immunohistochemical analysis of cellular infiltration.** The tissue sections were stained with rat anti-mouse F4/80, rat anti-mouse CD4 and CD8 antibodies to illustrate the containing of macrophages, CD4+ and CD8+ cells in skin transplantation with bm12 as the donor to B6 or Apn−/− as the recipients.
(TIF)

Figure S3 **Expression the cytokines mRNA in grafts.** Th1, Th17, co-stimulatory molecule PD-L1, APN and its receptors in grafts of bm12 as donor and B6 or Apn−/− as recipient by Q-PCR were analyzed. One sample was used in each B6 and Apn−/− group.
(TIF)

Figure S4 **A** Determination of serum FBS concentration used in MLRs containing splenocytes. **B** and **C** Effect of Compound C and SB203580 treatment on splenocyte apoptosis using Annexin V staining.
(TIF)

Figure S5 **Mixed lymphocytes reaction (MLR).** **A** Bone marrow derived dendritic cells from bm12 as the stimulator cells and T cells isolated from spleen of B6 or Apn−/− mice as the responder cells. The cells were mixed at different ratios and proliferation was measured with 3H-thymidine incorporation. **B** and **C** The maturation of dendritic cells from bm12 or B/c. Bone marrow cells were isolated and cultured for 7 days in present of IL-4 and GM-CSF, the cells was added LPS (100 µg/ml) for 1 more day to induce the maturation. CD80, CD86 and MHC class II were used as maturation markers.
(TIF)

Figure S6 **Expression of AdipoR1 and AdipoR2 on T cells isolated from spleen of B6 and Apn**−/− **mice.** The T cells were isolated with Dynal® CD4 Negative Isolation Kit and total RNA was extracted. The mRNA expression levels were determined by quantitative Q-PCR.
(TIF)

Table S1 **The primers used in Quantitative real-time PCR (Q-PCR).** The primers were synthesized by Invitrogen Hong Kong Ltd.
(DOC)

Table S2 **Serum levels of IFN-γ and IL-2 at 7 days post-operatively in mice following skin grafting.** n = 6 in each group (†*p*<0.01 compared to B6 to B6; ††*p*<0.001 compared to *Apn*−/− to *Apn*−/−; ‡‡*p*<0.01 compared to B6 to B6 and ‡*p*<0.05 compared to *Apn*−/− to *Apn*−/−).
(DOC)

Materials S1 (DOC)
