# Supplementary figures and images for: Adiponectin Mediated MHC Class II Mismatched Cardiac Graft Rejection in Mice Is IL-4 Dependent
Source: PLoS One. 2012 Nov 14;7(11):e48893. doi: 10.1371/journal.pone.0048893 (PMC3498365; doi:10.1371/journal.pone.0048893)

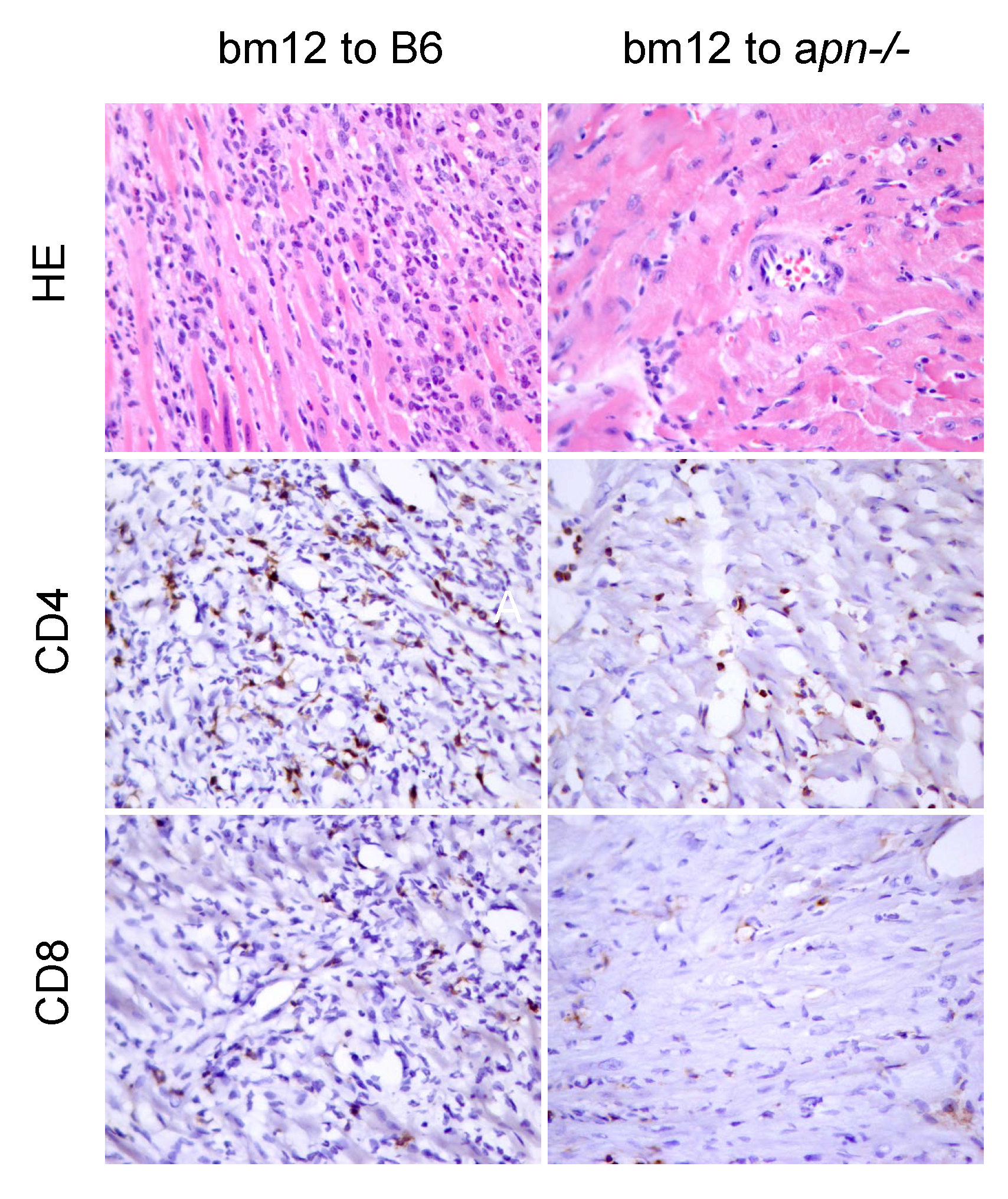

Supplement: Figure S1 — Immunohistochemical analysis of cardiac transplantation grafts. Sections were stained with Hematoxylin and Eosin (HE), rat anti-mouse CD4 and CD8 antibodies and bm12 to B6 grafts are compared to bm12 to APM−/− grafts. The samples were obtained from the grafts which the heart beating was stopped. (TIF) [file pone.0048893.s001.tif]

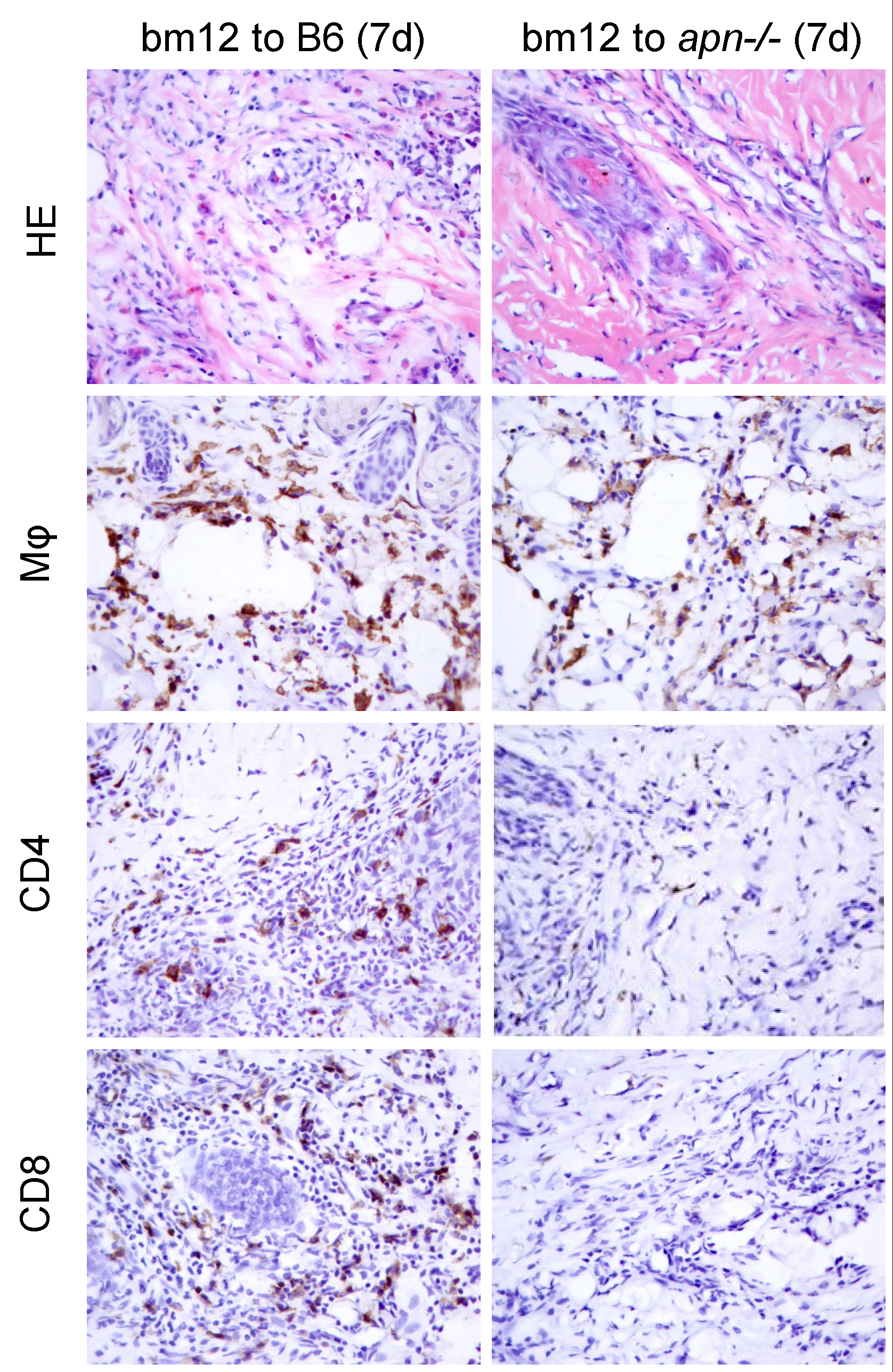

Supplement: Figure S2 — Immunohistochemical analysis of cellular infiltration. The tissue sections were stained with rat anti-mouse F4/80, rat anti-mouse CD4 and CD8 antibodies to illustrate the containing of macrophages, CD4+ and CD8+ cells in skin transplantation with bm12 as the donor to B6 or Apn−/− as the recipients. (TIF) [file pone.0048893.s002.tif]

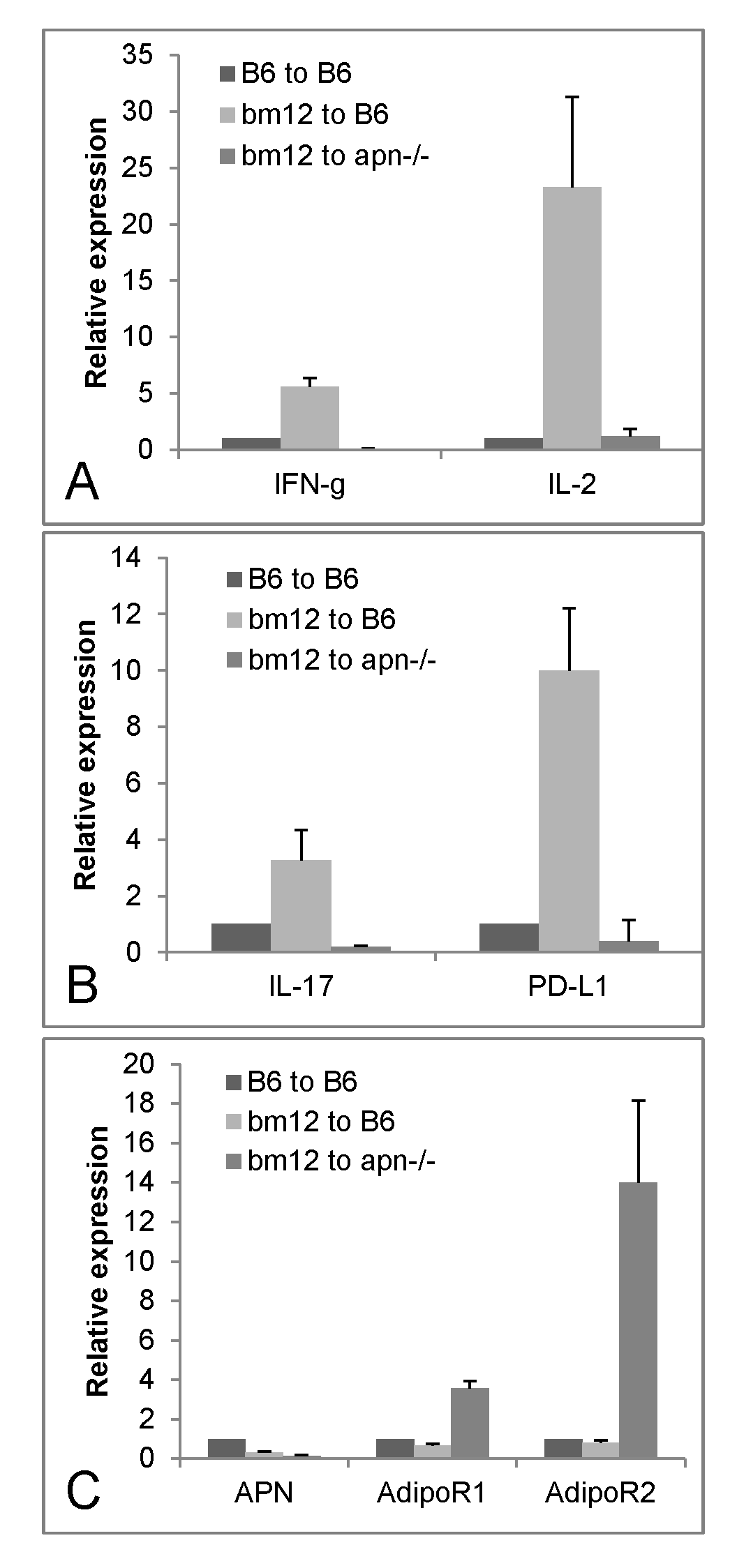

Supplement: Figure S3 — Expression the cytokines mRNA in grafts. Th1, Th17, co-stimulatory molecule PD-L1, APN and its receptors in grafts of bm12 as donor and B6 or Apn−/− as recipient by Q-PCR were analyzed. One sample was used in each B6 and Apn−/− group. (TIF) [file pone.0048893.s003.tif]

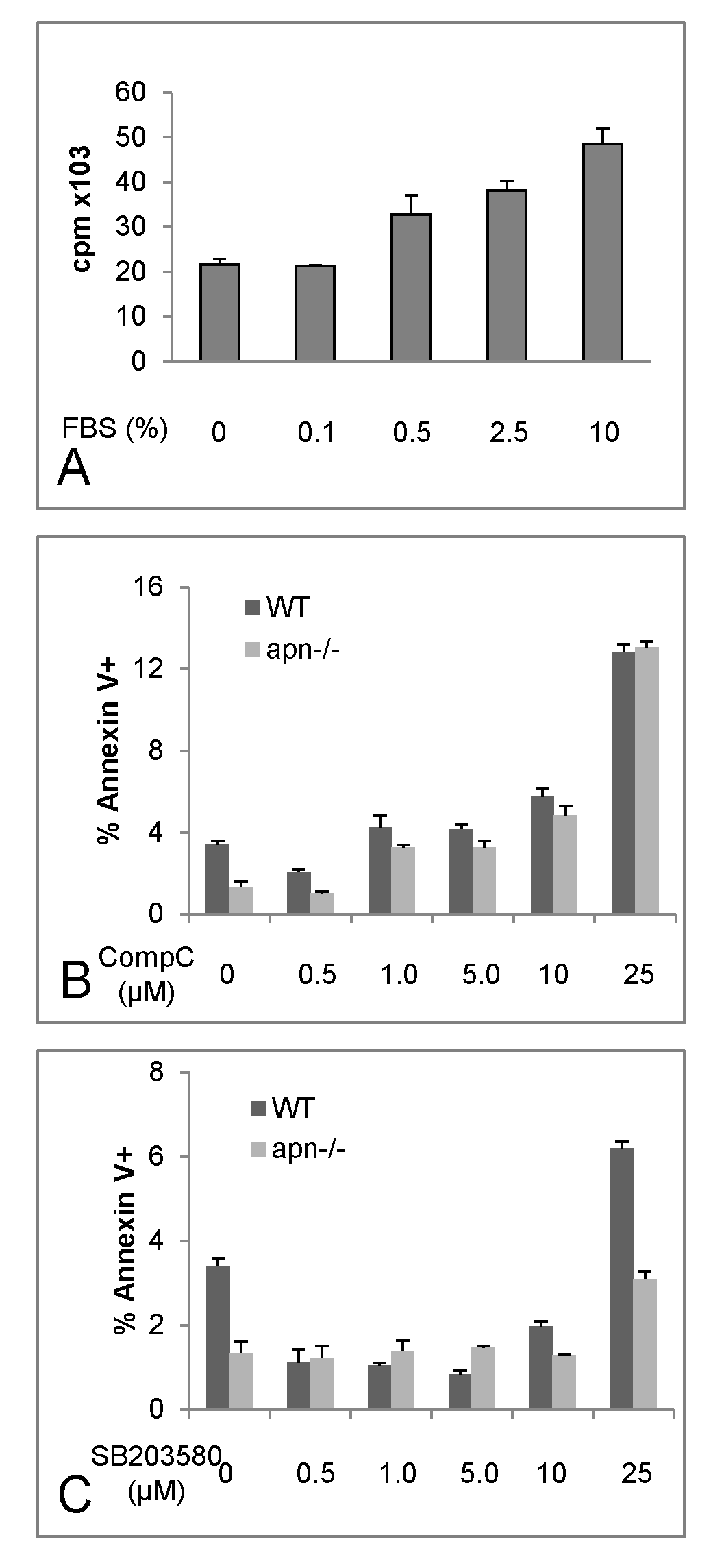

Supplement: Figure S4 — A Determination of serum FBS concentration used in MLRs containing splenocytes. B and C Effect of Compound C and SB203580 treatment on splenocyte apoptosis using Annexin V staining. (TIF) [file pone.0048893.s004.tif]

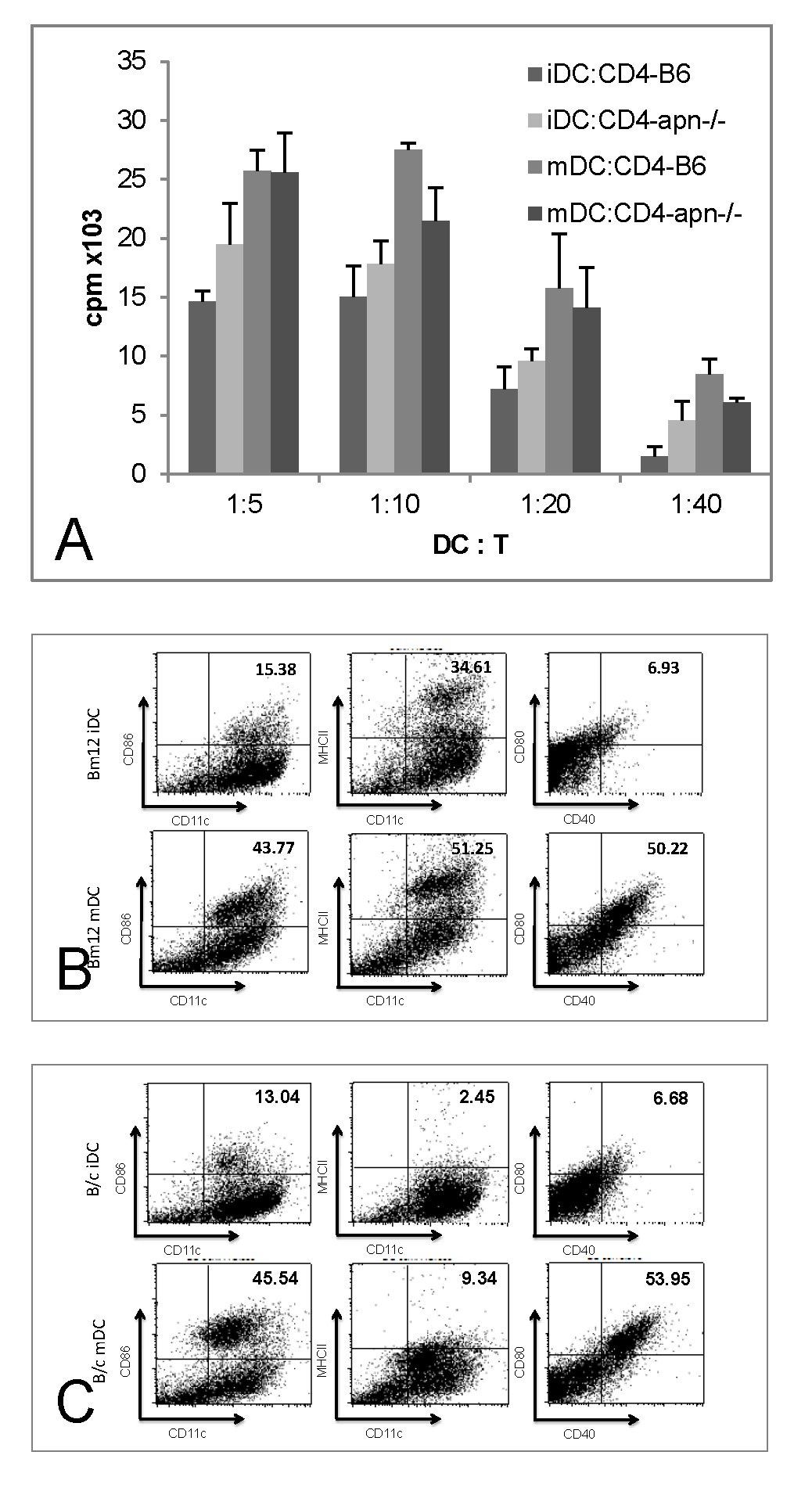

Supplement: Figure S5 — Mixed lymphocytes reaction (MLR). A Bone marrow derived dendritic cells from bm12 as the stimulator cells and T cells isolated from spleen of B6 or Apn−/− mice as the responder cells. The cells were mixed at different ratios and proliferation was measured with 3H-thymidine incorporation. B and C The maturation of dendritic cells from bm12 or B/c. Bone marrow cells were isolated and cultured for 7 days in present of IL-4 and GM-CSF, the cells was added LPS (100 µg/ml) for 1 more day to induce the maturation. CD80, CD86 and MHC class II were used as maturation markers. (TIF) [file pone.0048893.s005.tif]

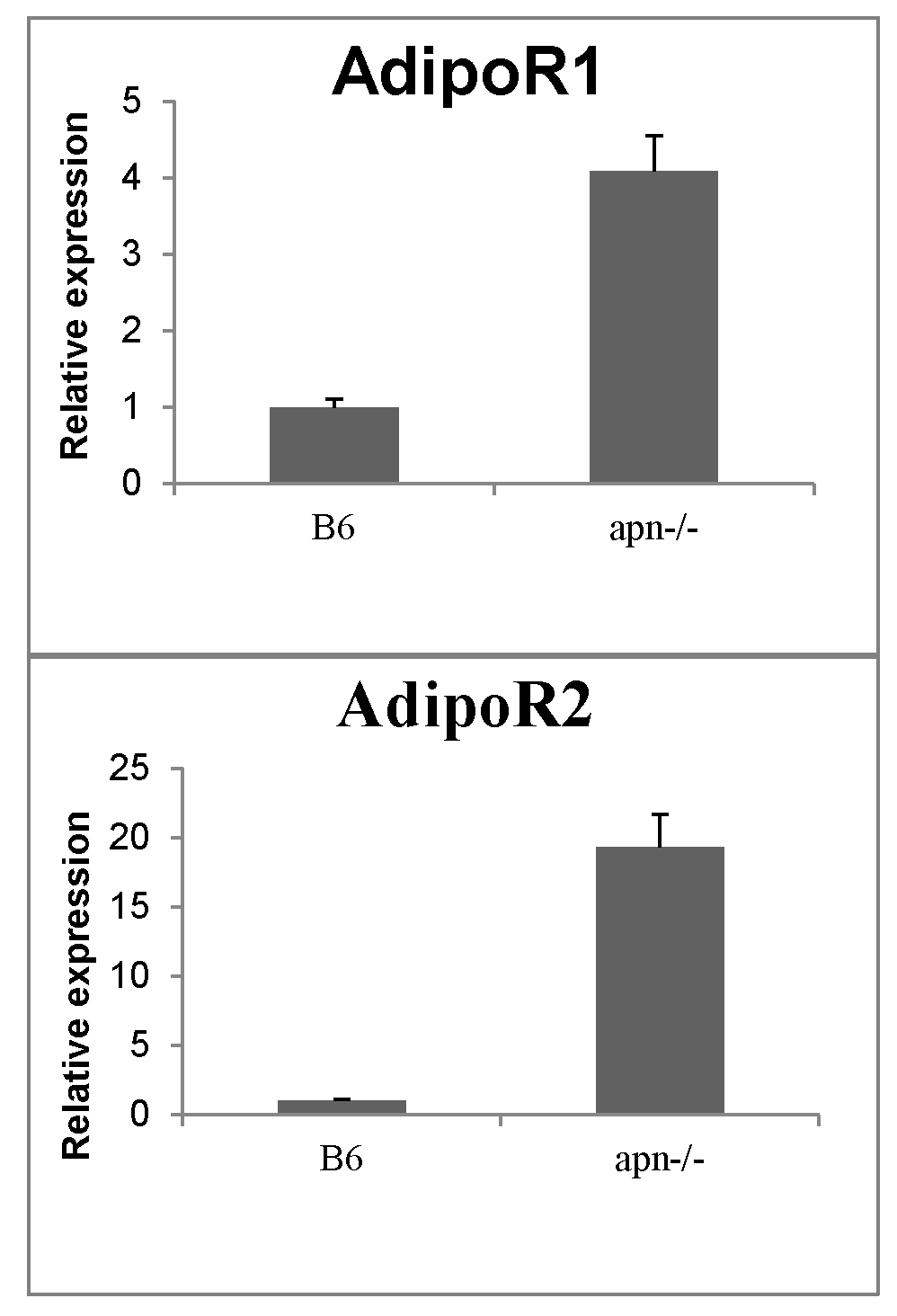

Supplement: Figure S6 — Expression of AdipoR1 and AdipoR2 on T cells isolated from spleen of B6 and Apn−/− mice. The T cells were isolated with Dynal® CD4 Negative Isolation Kit and total RNA was extracted. The mRNA expression levels were determined by quantitative Q-PCR. (TIF) [file pone.0048893.s006.tif]
