# Supplementary material for: Adiponectin Mediated MHC Class II Mismatched Cardiac Graft Rejection in Mice Is IL-4 Dependent
Source: PLoS One. 2012 Nov 14;7(11):e48893. doi: 10.1371/journal.pone.0048893 (PMC3498365; doi:10.1371/journal.pone.0048893)
Supplement: Table S1 — The primers used in Quantitative real-time PCR (Q-PCR). The primers were synthesized by Invitrogen Hong Kong Ltd. (DOC) [file pone.0048893.s007.doc]

**Table S1.** The primers used in Quantitative real-time PCR (Q-PCR). The primers were synthesized by Invitrogen Hong Kong Ltd.

| **Primers** | **Sequences** |
| --- | --- |
| Beta-actin | Forward: 5'- GGCTGTATTCCCCTCCATCG-3'  Reverse: 5'- CCAGTTGGTAACAATGCCATGT-3' |
| GAPDH | Forward: 5'-GTGTTCCTACCCCCAATGTG-3'  Reverse: 5'-TGAAGTCGCAGGAGACAACC-3' |
| AdipoR1 | Forward: 5'-ACGTTGGAGAGTCATCCCGTAT-3'  Reverse: 5'-CTCTGTGTGGATGCGGAAGAT-3' |
| AdipoR2 | Forward: 5’-GCCCAGCTTAGAGACACCTG-3’  Reverse: 5’-GCCTTCCCACACCTTACAAA-3’ |
| IFN-gamma | Forward: 5'-TCAAGTGGCATAGATGTGGAAGAA-3'  Reverse: 5'-TGGCTCTGCAGGATTTTCATG-3' |
| IL-2 | Forward: 5'-CCTGAGCAGGATGGAGAATTACA-3'  Reverse: 5'-TCCAGAACATGCCGCAGAG-3' |
| IL-4 | Forward: 5'-ACAGGAGAAGGGACGCCAT-3'  Reverse: 5'-GAAGCCCTACAGACGAGCTCA-3' |
| IL-5 | Forward: 5'-CCTGTCCCTACTCATAAAAATCACC-3'  Reverse: 5'-CCACTCTGTACTCATCACACCAA-3' |
| IL-10 | Forward: 5'-GGTTGCCAAGCCTTATCGGA-3'  Reverse: 5'-ACCTGCTCCACTGCCTTGCT-3' |
| IL-17 | Forward: 5'-CCTCAACCGTTCCACGTCAC-3'  Reverse: 5'-CCACACCCACCAGCATCTTC-3' |
| IL-12(P40) | Forward: 5'-GGAAGCACGGCAGCAGAATA-3'  Reverse: 5'-AACTTGAGGGAGAAGTAGGAATGG-3' |
| PD-L1 | Forward: 5'-TGCCCCATACCGCAAAATCAACC-3'  Reverse: 5'-GCTGGTCACATTGAGAAGCATCC-3' |
| siRNA-AdipoR1 (MSS231727) | 5'-CAU AGA AGU GGA CGA AAG CUG CUG C-3’ |
| siRNA-AdipoR2 (MSS229117) | 5'-AAG CCA AUC CGG UAG CAC AUC GUG A-3' |
