# Supplementary material for: Adiponectin Mediated MHC Class II Mismatched Cardiac Graft Rejection in Mice Is IL-4 Dependent
Source: PLoS One. 2012 Nov 14;7(11):e48893. doi: 10.1371/journal.pone.0048893 (PMC3498365; doi:10.1371/journal.pone.0048893)
Supplement: Table S2 — Serum levels of IFN-γ and IL-2 at 7 days post-operatively in mice following skin grafting. n = 6 in each group (†p<0.01 compared to B6 to B6; ††p<0.001 compared to Apn−/− to Apn−/−; ‡‡p<0.01 compared to B6 to B6 and ‡p<0.05 compared to Apn−/− to Apn−/−). (DOC) [file pone.0048893.s008.doc]

**Table S2.** Serum levels of IFN-γ and IL-2 at 7 days post-operatively in mice following skin grafting. n=6 in each group (†*p*<0.01 compared to B6 to B6; ††*p*<0.001 compared to *Apn-/-* to *Apn-/-*; ‡‡*p*<0.01 compared to B6 to B6 and ‡*p*<0.05 compared to *Apn-/-* to *Apn-/-*)

|  | **IFN-γ** | **IL-2** |
| --- | --- | --- |
| B6 to B6 | 28.85±9.09 | 11.41±2.34 |
| *Apn-/-* to *Apn-/-* | 33.90±14.04 | 8.86±1.60 |
| bm12 to B6 | 69.76±13.06†† | 20.55±4.94‡‡ |
| bm12 to *Apn-/-* | 88.55±10.92††† | 15.59±2.79‡ |
