# Supplementary material for: Adiponectin Mediated MHC Class II Mismatched Cardiac Graft Rejection in Mice Is IL-4 Dependent
Source: PLoS One. 2012 Nov 14;7(11):e48893. doi: 10.1371/journal.pone.0048893 (PMC3498365; doi:10.1371/journal.pone.0048893)
Supplement: Materials S1 — (DOC) [file pone.0048893.s009.doc]

**Materials S1**

Flow cytometer related antibodies, rat anti-mouse CD40, CD86, MHC class II and CD11c were purchase from BD Biosciences (BD Biosciences, San Jose, CA). The antibodies, rat anti-mouse CD4, CD8 for immunostaining, ELISA kit for IL-4, rat-anti mouse neutralizing antibody anti-IL-4 and mouse recombinant IL-4 were obtained from BioLegend (BioLegend San Diego, CA). Reverse transcription kit was obtained from Promega (Promega Corp. Madison, WI The Q-PCR reagents were from Applied Biosystems (Applied Biosystems, Foster City, CA). The chemical reagents for preparing Carbol-chromotrope, Victoria blue and Masson’s trichrome staining solution, SB203580 and Metformin were obtained from Sigma-Aldrich Corp. St. Louis, MO, USA. Compound C was obtained from Merck KGaA, Darmstadt, Germany. Mouse full length recombinant adiponectin was obtained from Dr. Aimin Xu, Department of Medicine, the University of Hong Kong. siRNA for Adiponectin receptor 1 and 2 and RNAi duplex-Lipofectamine™ RNAiMAX were purchased from Invitrogen (Invitrogen HK Ltd, NT, Hong Kong). Antibodies for western blot, Adiponectin receptor 1 and 2 were from Alpha Diagnostic, San Antonio, TX, USA. GATA-3 was from eBioscience, San Diego, CA, USA. STAT-6 and phospho-STAT6 were from Santa Cruz (Santa Cruz, CA. USA). p38 and phospho-p28 were purchased from cell signaling (Cell Signaling Technology, Inc., Danvers, MA USA). Dynal® CD4 Negative Isolation Kit was obtained from Invitrogen (Invitrogen Dynal AS). Heparin was purchased from Leo, Laboratories Ltd., UK. For animal analgesic or tranquilizing drugs used in operation, the Pentobarbital Sodium (40-65mg/kg , i.p.) was used.
